# European survey on national harmonization in clinical research

**DOI:** 10.1002/lrh2.10220

**Published:** 2020-02-21

**Authors:** Annette Magnin, Valentina Cabral Iversen, Gonzalo Calvo, Beata Čečetková, Ola Dale, Regina Demlová, György Blaskó, Fionnuala Keane, Gabor L. Kovacs, Claire Levy‐Marchal, Emilia C. Monteiro, Lucia Palmisano, Daniel Pella, Antonio Portolés, Olivier Rascol, Caecilia Schmid, Fabian Tay, Heiko von der Leyen, Christian Ohmann

**Affiliations:** ^1^ SCTO—Swiss Clinical Trial Organisation Bern Switzerland; ^2^ NorCRIN—Norwegian Clinical Research Infrastructure Network Trondheim Norway; ^3^ SCReN—Spanish Clinical Research Network Madrid Spain; ^4^ SLOVACRIN—Slovak Clinical Research Infrastructure Network Košice Slovakia; ^5^ CZECRIN—Czech Clinical Research Infrastructure Network Brno Czech Republic; ^6^ HECRIN—Hungarian Clinical Research Infrastructure Network Pécs Hungary; ^7^ HRB CRCI—Health Research board, Clinical Research Coordination Dublin Ireland; ^8^ F‐CRIN—French Clinical Research Infrastructure Network Toulouse France; ^9^ PtCRIN—Portuguese Clinical Research Infrastructure Network Lisbon Portugal; ^10^ ItaCRIN—Italian Clinical Research Infrastructure Network Rome Italy; ^11^ KKSN—Netzwerk der Koordinierungszentren für Klinische Studien Berlin Germany; ^12^ ECRIN—European Clinical Research Infrastructure Network Paris France

**Keywords:** clinical research, clinical study, clinical trial, ECRIN, EU 536/2014, harmonization of processes, survey

## Abstract

**Background:**

Clinical trials remain key to the development of evidence‐based medical practice. However, they are becoming increasingly complex, mainly in a multinational setting. To address these challenges, the European Union (EU) adopted the Clinical Trial Regulation EU No. 536/2014 (CTR). Once in force, the CTR will lead to more consistent rules and simplification of procedures for conducting clinical trials throughout the EU. Existing harmonization initiatives and “research infrastructures” for clinical trials may facilitate this process. This publication offers a snapshot of the current level of harmonization activities in academic clinical research in Europe.

**Methods:**

A survey was performed among the member and observer countries of the European Clinical Research Infrastructure Network (ECRIN), using a standardized questionnaire. Three rounds of data collection were performed to maximize completeness and comparability of the received answers. The survey aimed to describe the harmonization of academic clinical research processes at national level, to facilitate the exchange of expertise and experience among countries, and to identify new fields of action.

**Results:**

Most scientific partners already have in place various working groups and harmonization activities at national level. Furthermore, they are involved in and open to sharing their know‐how and documents. Since harmonization was mainly a bottom‐up approach up until now, the extent and topics dealt with are diverse and there is only little cross‐networking and cross‐country exchange so far.

**Conclusions:**

Currently, the ECRIN member countries offer a very solid base and collaborative spirit for further aligning processes and exchanging best practices for clinical research in Europe. They can support a smooth implementation of the EU CTR and may act as single contact with consolidated expertise in a country.

## 1 INTRODUCTION

Clinical trials remain key to the development of evidence‐based medical practice. Over the years, there has been a growing trend to perform large‐scale clinical trials across borders, not only in industry but in academic settings as well. Multinational collaboration brings many advantages for all types of clinical trials. Trial participant recruitment is faster, and the results of the trial are more generally applicable. On the other hand, multinational clinical trials are significantly more complex to perform than national ones due in particular to the difficulties arising from the diversity of legal frameworks and operational practices. This is particularly true for the conduct of multinational trials initiated by academic institutions, which often do not have well‐developed on‐site management support. As a consequence, only 3% of academic trials are international (whereas 30% of industry trials are international).^1^ In addition, the number of clinical trials, particularly those initiated by academic investigators, has been falling in recent years in many regions, including the European Union (EU).^2^

In order to improve this situation, the EU adopted the Clinical Trial Regulation EU No. 536/2014 (CTR^3^). Once in force, the CTR will lead to harmonized rules for conducting clinical trials throughout the EU. This will increase the efficiency of all trials in Europe with the greatest benefit for those conducted in multiple European Member States. It aims, among other things, to improve collaboration, information‐sharing, and decision‐making between and within Member States.^3^ The Heads of Medicines Agencies (HMA) established the Clinical Trials Facilitation Group (CTFG) in order to harmonize activities across Member States.^4^ To ease the implementation of the CTR, pilot projects have begun at national level in some countries. These national pilot projects have focused on coordinated assessment by national competent authorities (NCAs) and ethics committees (ECs) within a single Member State.^4^

To what extent does harmonization of clinical research already exist in Europe? Through the Voluntary Harmonization Procedure (VHP), which was implemented in 2009 by the HMA, preliminary experience has been gained for the harmonization of clinical trial applications in particular (through mutual assessments). The CTR will further change and harmonize many processes related to multinational clinical trials, and these processes need to be adopted by existing “research infrastructures” and institutions. The term “research infrastructure” is used here to mean an organization that provides facilities, resources, or related services to researchers to enable top‐level scientific research; in the particular context of this article, we refer specifically to “infrastructures” that support multinational clinical research in Europe. We believe that these types of infrastructures, as well as existing harmonization processes (at national level), may be relevant for the implementation of the CTR (and beyond). As such, this paper aims to provide a snapshot of the current level of harmonization activities in clinical research; it also provides an overview of the current/potential role of infrastructures such as the European Clinical Research Infrastructure Network (ECRIN) to align harmonization processes between countries, in accordance with the expectations or requirements of the CTR (see also Appendix S1 for major changes under the CTR).

ECRIN is a public, nonprofit organization that links scientific partners and networks across Europe in order to facilitate multinational clinical research.^5^ Established in 2004, ECRIN was awarded the legal status of a “European Research Infrastructure Consortium” (ERIC) in 2013. ECRIN's scientific partners are national networks that are composed of academic clinical research centers (CRCs) or clinical trial units (CTUs) with a national coordinating center (Table 1). As per the ECRIN‐ERIC statutes, the scientific partners must have developed shared tools, procedures, and practices to facilitate multicenter studies and have reached a “critical mass” in terms of competency.^6^ ECRIN's scientific partners are involved in various harmonization activities at national level and beyond. To identify the scope of these activities, a survey was performed in ECRIN member and observer countries. The findings of the survey are also intended to support the identification of new fields of action for ECRIN to increase the quality and efficiency of multinational clinical research.

**TABLE 1:** National clinical research networks belonging to ECRIN

Country	ECRIN Scientific Partner
Czech Republic	CZECRIN—Czech Clinical Research Infrastructure Network https://www.czecrin.cz/
France	F‐CRIN—French Clinical Research Infrastructure Network https://www.fcrin.org/
Germany	KKSN—Netzwerk der Koordinierungszentren für Klinische Studien https://www.kks‐netzwerk.de/
Hungary	HECRIN—Hungarian Clinical Research Infrastructure Network https://hecrin.pte.hu/
Ireland	HRB CRCI—Health Research board, Clinical Research Coordination Ireland https://www.hrb‐crci.ie/
Italy	ISS—Istituto Superiore di Sanita/ItaCRIN—Italian Clinical Research Infrastructure Network https://www.itacrin.it/
Norway	NorCRIN—Norwegian Clinical Research Infrastructure Networkhttps://www.norcrin.no/
Portugal	PtCRIN—Portuguese Clinical Research Infrastructure Network http://www.ptcrin.pt/
Slovakia^a^	SLOVACRIN—Slovak Clinical Research Infrastructure Network https://slovacrin.sk/
Spain	SCReN—Spanish Clinical Research Network https://www.scren.es/en/scren.php
Switzerland^a^	SCTO—Swiss Clinical Trial Organization https://www.scto.ch/en/news.html

*Note*. Link to short descriptions of the ECRIN scientific partners (members and observers) listed above: https://www.ecrin.org/who‐we‐are/members‐observers

a Observer.

## 2 RESEARCH INTERESTS

Research interests of the survey cover a status report on harmonization activities and the identification of possibilities to improve.

- To identify the current level of harmonization activities in clinical research prior to implementation of the EU CTR
- To enable synergies by facilitating the exchange of expertise and experience in national harmonization in clinical research
- To identify new fields of action for ECRIN to increase the quality and efficiency of multinational clinical research

## 3 METHODS

A survey was performed among ECRIN's national scientific partners in its member and observer countries (see Table 1) using a standardized, open‐ended questionnaire.

Background information about ECRIN is given in Appendix S2.

In Table 2, basic data about the National Clinical Research Networks belonging to ECRIN are summarized.

**TABLE 2:** Summary information about national clinical research networks belonging to ECRIN^5^

Country	ECRIN Scientific Partner	Year Established	Annual Budget in 2019 (€)	Number of Institutions in each network in 2019	Number of Ongoing Multicenter Trials in 2019, where a CTU of the National Network is Involved	Number of ECRIN Certified Data Centers in 2019	Staff Number at Headquarters in 2019 (FTE)	Comments
Czech Republic	CZECRIN—Czech Clinical Research Infrastructure Network	2014	72 500	9	21	0	2,5	Staff includes the employees of the national hub
France	F‐CRIN—French Clinical Research Infrastructure Network	2011	1.1 M	8	124	2	± 10	
Germany	KKSN—Netzwerk der Koordinierungszentren für Klinische Studien	2004	520 000 (planned annual budget for 2020 after employing all EuCos at the national hub)	24	multicenter trials: 608 all trials: 884	6	4,41 (beginning 2020)	Expenses for EuCos (approx. 160 000 €/year) are included in the planned annual budget; beginning of 2020 all EuCos (1,91 FTE) are employees of the national hub
Hungary	HECRIN—Hungarian Clinical Research Infrastructure Network	2006*	77 842	17	8 (CTU) 10 (CRO)	0	4	*2006–2014 HECRIN Committee of the HungarianMedical Research Council 2014—HECRIN Consortium (full member of ECRIN)
Ireland	HRB CRCI—Health Research board, Clinical Research Coordination Ireland	2015	786 215	7 Institutions 8 clinical research centers	Multicenter, interventional trials ongoing in 2019 across all 8 centers 223	0	5.6	funding for the HRB CRCI central office only. Includes ECRIN EuCo funding (1FTE).
Italy	ItaCRIN—Italian Clinical Research Infrastructure Network	2013 (sig–nature of the MoU)	324 206 (annual fee) +50 000 extra expenses	9	18	3	2,1	Staff includes all employees of the national hub (dedicating different proportions of their time). Based on these proportions, the expense for salaries is 116.175 euros
Norway	NorCRIN—Norwegian Clinical Research Infrastructure Network	2012	650 000	6	143#	1	2.2	Data center certified 2019 Oslo
Portugal	PtCRIN—Portuguese Clinical Research Infrastructure Network	2014	80 290 plus 1,3 FTE in kind	9 integrated members including 5 CTUs	6	1	2,3	
Slovakia(observer)	SLOVACRIN—Slovak Clinical Research Infrastructure Network	2018	80 000*	6	4	0	2,5	*budget under Medical Faculty of Pavol Josef Safarik University without national funding so far, EuCos and other collaborators included in budget estimation
Spain	SCReN—Spanish Clinical Research Network	2009	600 000*	31	136**	0	6	*including Human Resources, costs of services, coordination, formation, etc **about 21 of them are ECRIN
Switzerland(observer)	SCTO—Swiss Clinical Trial Organization	2009	1 M (SCTO EO)	6	447 (Multicenter, national & multinational) Numbers refer to 2018—the 2019 data are available in Feb 20)	(1) underwent audit	6.7	SCTO EO coordinates a pediatric RI as well and includes ECRIN EuCo.

Abbreviations: CRC, Clinical Research Centre; CTU, Clinical Trial Unit; EuCo, European Correspondent; FTE, Full time Equivalent; MoU, Memorandum of Understanding.

Three rounds of data collection were performed between April 2018 and January 2019 to maximize completeness and comparability of the answers received. The detailed procedure of the data collection rounds is already described by Magnin et al.^7^

The survey consisted of two parts, one dealing with national harmonization activities and the other with national training activities. The results of the latter were published in the *Trials Journal*.^7^ This report summarizes the results of the survey describing the harmonization of academic clinical research processes at national level. The questionnaire is included in Appendix S3.

## 4 RESULTS

All ECRIN member and observer countries participated in the survey. Table 3 gives an overview of the responses on national harmonization activities, as provided by ECRIN's different scientific partner networks.

**TABLE 3:** Summary of answers to the question: “To what extent do you work on the harmonization of clinical research processes at national level?”

Harmonization activity	Country										
	CZE	FRA	DEU	HUN	IRL	ITA^a^	NOR	POR	SVK	ESP	CHE
Working groups	X	X	X	X	X		X	X	X	X	X
Consulting	X	X	X	X	X		X	X	X	X	X
SOPs	X		X				X	X		X	X
Templates, tools	X		X		X		X		X	X	X
Services	X	X	X		X		X				
Curricula for CR staff	X		X				(X)	X			X
Other activities			X	X	X		X	X			

Abbreviations: CZE Czech Republic, FRA France, DEU Germany, HUN Hungary, IRE Ireland, ITA Italy, NOR Norway, POR Portugal, SVK Slovakia, ESP Spain, CHE Switzerland; SOPs, standard operating procedures; CR, clinical research; X, available; (X), planned.

a In construction phase.

### 4.1 Working groups

All but one of the scientific partners have established (or participate in national) working groups (WGs) in order to harmonize practices. In many cases, there is a systematic approach for collaboration. Depending on the topic, collaboration may also be opportunity driven. WGs (or initiatives) by country are as follows:

- Czech Republic: “Working Committee for Clinical Trials at the Ministry of Health”
- France: WG on harmonization of practices with French industry
- Germany: numerous WGs with all relevant stakeholders to implement common standards in relevant fields
- Hungary: Human Resource Development Operational Program (HRDOP) and its subprojects^8^
- Ireland: participation in various WGs through Health Research Board—Clinical Research Coordination Ireland (HRB‐CRCI)
- Norway: Consults with the medicinal authorities once a year and provides consulting services on most aspects of a clinical trial, primarily good clinical practice (GCP), contractual work, budget, data management, and monitoring
- Portugal: WGs on trial participant reimbursement, implementation of General Data Protection Regulation (GDPR), patient access to trial information, human resource recruitment for CRCs, and translation of medical device standards
- Slovakia: initiative to establish a national contract template for clinical trials and training program for CTU staff
- Spain: development and implementation of the new clinical trial legislation
- Switzerland: 8 expert WGs (ie, thematic platforms) of the network to streamline clinical research and active participation in national WGs^9^

### 4.2 Consulting

Consultation is provided in 10 countries and may be initiated by networks or asked for by national partners. In each country, this covers the following:

- Czech Republic: consultation on specific study documents and legal documents (eg, adoption of CT directive initiated by the national regulatory agency SUKL)^10^
- France: consultation with external bodies (eg, Ministry of Health [MOH])
- Germany: consultation on laws and regulations (eg, leading role in radiation protection law, implementation of GDPR, national implementation on medical device regulation [MDR])
- Hungary: consultation is provided as part of the HRDOP^8^
- Ireland: consulting on any relevant issues
- Norway: consulting within the above‐mentioned WGs
- Portugal, Spain, and Switzerland: consultation on laws and regulations in collaboration with national bodies (eg, human research act, biobank regulations, and type of intervention and submission process)
- Slovakia: a national advisory group of all experts and stakeholders in clinical research is planned

It should be stated here that consultancy may have been interpreted differently between the national partners (see Section 5).

### 4.3 Standard operating procedures

Six countries are working on or have provided harmonized standard operating procedures (SOPs) (for external and network internal use), which have to be adapted to local processes (Czech Republic, Germany, Norway, Spain). Portugal developed SOPs for monitoring for newly established CTUs and disseminates SOPs developed by international organizations; furthermore, their WGs will issue additional SOPs. In Switzerland, SOPs are developed within the thematic platforms; furthermore, an overarching Guideline for Good Operational Practices is available for free.^11^

### 4.4 Templates/tools

Templates and tools have been developed by seven scientific partners (for network internal purposes and general use). This involves, for example, templates for national grant proposals in Czech Republic (network internal use); templates for GDPR‐compliant patient sheets in Germany; standard Clinical Trial Agreements (CTAs) and costing templates in Ireland; several templates in Switzerland, e.g. a tool for risk‐adapted monitoring,^12^ data management guidelines, as well as many templates in collaboration with swissethics.^13^

### 4.5 Services

The Czech network is responsible for the national harmonization of services (not further specified). The French network is involved in a national “train the trainer” program for clinical research. In Germany, the output from consulting and WGs sometimes results in services. Ireland reported the provision of services covering streamlined feasibility services, pharmacovigilance (PV) services, and management of a central contact point for Ireland. Norway supports the pharmaceutical industry and academic researchers by streamlining processes.^14^

### 4.6 Other activities and general remarks

In Ireland, a mutual recognition scheme for quality management (QM) for the HRB CRCI network and a clinical research program for the country have been established. In Hungary, the HRDOP was created to develop and internationalize a thematic network for clinical research, with the involvement of HECRIN. In Portugal, a course for hospital managers on CRC organization was offered in collaboration with the Portuguese Association of Hospital Managers (APAH) and the Portuguese Association of the Pharmaceutical Industry (APIFARMA). For Norway, see above (under WGs).

The mandate of the French network was initially more focused on bringing different players together to improve efficiency (through “structuring”). The better the collaboration between actors, the more national harmonization can be seen. The KKS‐Network in Germany is contributing to a broad range of issues related to clinical research.

In Italy, the national context is going to change due to a new law that will provide rules on several aspects of clinical research, including educational programs for operators and requirements for the clinical sites involved in phases I to IV clinical trials.^15^

As of December 2018, a new ERASMUS^+^ project (“Curriculum Development of Human Clinical Trials for the Next Generation Biomedical Students,” CONSCIOUS) has been initiated. The project leader is HECRIN, and the scientific partners are CZECRIN, PtCRIN, F‐CRIN, and MMI Clinical Research Development Ireland. The general objective of the CONSCIOUS project is to tackle the skill gaps and mismatches related to European‐level clinical trial professionals through curriculum development and preparation of e‐learning material for the career development of biomedical (medical, pharmacy, clinical research master) students.

The current state of harmonization efforts in training and curricula for clinical research is described in Magnin et al.^7^

### 4.7 Sharing templates/documents/recommendations

All countries share templates/documents/recommendations developed in their network, except for Italy, which shares only within ECRIN‐related studies. In addition, some countries provide external access upon request (Portugal), or on a cases‐by‐case basis (Germany), or for specific documents (Switzerland). Open and free access is provided by Ireland (with a disclaimer), and this will be the case for Norway, Slovakia, and Switzerland in the future.

Appendix S4 provides a noncomprehensive overview with useful links provided by the scientific partners. Furthermore, ECRIN harmonization activities and corresponding links are listed as well. To establish a more comprehensive future repository as planned by ECRIN, more conceptual work and resources are required, including a process to maintain the information (see also Section 5).

### 4.8 Local/national certification programs for clinical research

The majority of countries offer no local/national certification programs for aspects of clinical research (Czech Republic, Germany, Hungary, Norway, Switzerland). In France, researchers who attended a 6‐day training course for educational development of clinical investigation trainers received a certificate. In Italy, certification is necessary for phase 1 units, in Spain, the latter certification is available, but not mandatory, and in Portugal, investigators and research teams are certified in GCP. In Ireland, HRB‐CRCI has established a mutual recognition scheme for QMS, issuing a letter. In addition, there is a graduate certificate in clinical research from the University College Dublin (UCD). In Germany and Switzerland, audits (but not certification) are a prerequisite for membership. The answers are summarized in Table 4. It should be noted that there is some diversity of interpretation and use of terms related to certification (see Section 5).

**TABLE 4:** Answers to the question “Do you have or offer local/national certification programs for aspects of clinical research?”

Country	Do you have or offer local/national certification programs for aspects of clinical research?
Czech Republic	No, but currently discussing within the “Working Committee for Clinical Trials at the Ministry of Health” (planned start date: May 2019)
France	Sites performing phase I/first‐in‐man trials need certification. Evaluation of disease‐targeted investigator networks undergoing a specific selection program results in labelling, not certification. Certification of trainers attending the training course for educational development of clinical investigation trainers.^16^
Germany	Audits for new KKS members are a prerequisite for membership. Also teaching curricula certification. Target group: within network.
Hungary	No. The National Institute of Pharmacy and Nutrition (OGYÉI) is the authority: they offer and perform the local certification program. Consortium members organize professional conferences, where it is possible to complete the GCP course for all interested (external and internal) parties. As the part of the HRDOP project (Education development subproject) we are also working on curriculum development. The teaching materials developed for clinical trial staff (study coordinator, study nurse, investigator, sub‐investigator) will be available not only within the universities but also externally.
Ireland	Yes, on completion of the Mutual Recognition Scheme for QMS, the centers are issued a letter of completion (mutual recognition) from HRB‐CRCI. ICH GCP training for Investigational Medicinal Products (IMPs) and medical devices is carried out by the Clinical Research Facilities and Centers (CRF/C's) in the network and certificates of completion are issued by the centers. The target groups for these courses are the CRF/C's and their staff as well as the investigator teams running the trials from the hospitals engaged with the CRF/C's for clinical research. Individual centers offer a range of different clinical research training courses in addition to those listed above.
Italy	Certification is required for clinical research organizations (CROs) (for any kind of study) and for clinical sites only if involved in phase I studies. In both cases they self‐certify their activities. Inspections by the Italian Competent Authority will follow. We do not offer any certification programs.
Norway	Planned: certification program for study nurses/coordinators. In discussion: certification “light” for early phase units.
Portugal	Clinical Investigator Certificate^17^ is an e‐learning‐based course of 16h (Level I) and 40h (Level II) training. The program is based on the syllabus published by PharmaTrain and ECRIN (Boeynaems et al^18^). A GCP certificate is obtained by clinical research staff and investigators after passing the final exam. One CTU is certified under ISO 9001:2015 rules that also certifies recruitment centers in ophthalmology (national and international)
Slovakia^a^	No
Spain	Some CTUs are: Certified under ISO 9001:2015 rules Certified for GCP compliance (Phase I units)
Switzerland^a^	No certification programs for CTUs and research institutes (however, audits are a prerequisite for new members). Trainings and certificates for clinical research professionals.

a Observer.

### 4.9 Influence on national harmonization regarding clinical research

A broad spectrum of ratings of the influence of ECRIN scientific partners on harmonization of clinical research in their country was observed. The influence was rated as “high” in three countries, “moderate” in two countries, “low” in three countries, and “very low” in one country. One country reported “low” in harmonization but “very high” in structuring and one “moderate to high.” One country that rated the influence as “low” stated that more governmental support is needed for the implementation of harmonization. Another rating was “moderate” because the network is only 3 years in operation; however, it is already collaborating with the major stakeholders. In the country with a rating of “moderate to high,” the national network has become a recognized stakeholder for academic research; however, so far, there was little involvement in the legislative process.

### 4.10 Perception as facilitator for clinical research

All of ECRIN's scientific partners are perceived as facilitators rather than an additional source of bureaucracy. Three countries added that CTUs might be perceived as increasing administrative workload, since they continue to ask researchers to comply with the regulations (for the sake of quality but resulting in more paperwork).

## 5 DISCUSSION

Almost all ECRIN members are involved in national harmonization activities and are open to sharing their know‐how and documents. However, since harmonization was mainly a bottom‐up approach up until now, the extent and topics dealt with are diverse and there is little cross‐network exchange so far.

Currently, ECRIN member and observer countries offer a very good foundation and collaborative spirit to further align processes and exchange best practices for clinical research in Europe. This will play a key role not only for competitiveness in clinical research but also for building transnational awareness. Even though there is little systematic approach for (top‐down) strategic and overarching considerations, the already existing network will facilitate a stronger, future, EU cross‐border exchange. ECRIN members and observers can support a smooth implementation of the EU CTR and may act as a single contact with consolidated expertise in a country (see Appendix S2). By further coordinating its members/observers, and with the CTR providing a general framework, ECRIN will be able to further promote harmonization activities across Europe. This is contingent, however, on sustainable funding for ECRIN's national scientific partners. Governments should be aware of the critical need for funding for this type of activity as well as its excellent potential return on investment.

Furthermore, as a clinical *research* infrastructure, ECRIN's (and its scientific partners') services are not limited to clinical *trials* but offered for any kind of clinical research (this may, as an example, include noninterventional clinical research, reuse of biosamples, and patient data). The potential for multinational harmonization through clinical research infrastructures may even be higher in those areas of clinical research that are not regulated under the CTR.

### 5.1 Use of the survey results as an inventory to exchange experience and expertise:

A significant amount of experience and expertise is available within the ECRIN member and observer countries, yet knowledge exchange could be improved. Systematic exchange between countries may challenge and enrich national views and provide exposure to the broader European picture and vice versa. Lastly, the access to information, documents, and expertise should lead to a mutual benefit for all members, avoiding duplication of efforts and a waste of taxpayer money. Examples for such an exchange of expertise are how to handle GDPR, general consent processes, big data and data governance at institutional level, impact of personalized health on clinical research, and “horizon scanning” at European level. Furthermore, the interaction between ECRIN partners may facilitate the understanding of specific clinical trial–related regulatory processes, which, according to regulation 536/2014, have to be implemented at the national level by Member States. ECRIN and its scientific partners may also be an indispensable partner for the EU (respectively the responsible taskforces) in continuously harmonizing and implementing processes at European level once the CTR comes into force.

### 5.2 Further new fields of action for ECRIN to increase the quality and efficiency of multinational clinical research

- Certification: As a first successful model, ECRIN has implemented the certification of data centers.^19^ As a next step, certification in other areas could be envisaged (eg, pharmacovigilance or phase 1 units) to facilitate collaboration between partners in a multinational clinical trial.
- Closer collaboration with other “research infrastructures” could be an aim for the certification of biobanks, for example.
- Collaboration with different international registries for clinical trials in order to align content and improve comparability (eg, to better identify funding sources and data ownership).^20^
- Transnational implementation of the Erasmus^+^ CONSCIOUS project on innovative educational methodology will provide a new tool for many countries to standardize clinical trials and increase their efficiency at EU level and worldwide.
- ECRIN national scientific partners need to work towards more visibility at national level to foster quality and efficiency of multinational clinical research.^21^
- Collaboration and agreements to facilitate cross‐border data sharing through harmonized processes and best practices (citation: “The more broadly adopted standards (for data, metadata, models, and terminology), the easier the sharing of data and communication of meaning along with that data”).^22^
- A similar survey performed on a regular basis will make it possible to evaluate the “structuring effect” of ECRIN over time.

### 5.3 Limitations of the survey

Data collection was limited to 11 ECRIN member and observer countries through the coordinating units of the national scientific partners. The ECRIN partners served as a convenient and representative sample. However, we cannot exclude other harmonization activities not known to the networks. In addition, the national networks of the member/observer countries are all organized differently, ranging from few units providing services nationwide to a network of up to 30 CTUs across the country.^23^ As a consequence, the information is very heterogeneous and may not be complete in all cases and thus may not be representative for the entire EU.

In addition, diversity of interpretation and use of terms was identified in the survey as, for example, the “process of certification.” Does it mean a very rigorous process like ISO certification or does it refer to the simpler process of getting, for example, a certificate for training? Also “consultancy” was interpreted differently: consultancy as a core business of CTUs for researchers vs acting as experts for different bodies and authorities. For future surveys, such expressions should be better defined or provided in closed‐ended questions.

Nevertheless, the survey has been performed in three rounds with stepwise improvement of the information and face‐to‐face discussions between the survey participants and thus gives a good overview on harmonization activities in the participating countries. Just recently, Poland joined ECRIN as an observer, and hence, future updates will be even more comprehensive.

## CONFLICT OF INTEREST

The authors affirm that they have no conflicts of interest to disclose. This survey was conducted with no external funding or specific internal funding and was done within the usual employment contracts.

**Abbreviations**
CRC: clinical research center
CT: clinical trial
CTA: clinical trial agreement
CTFG: clinical trials facilitation group
CTR: clinical trial regulation
CTU: clinical trial unit
DM: data management
eCRF: electronic case report form
ECRIN: European Clinical Research Infrastructure Network
EU: European Union
GCP: good clinical practice
GDPR: General Data Protection Rule
HMA: Head of Medicines Agencies
HRB‐CRCI: Health Research Board Clinical Research Coordination Ireland
HRDOP: Human Resource Development Operational Program
ISO: International Organization for Standardization
MDR: Medical Device Regulation
MOH: Ministry of Health
NC: Network Committee
NCA: National Competent Authorities
PV: pharmacovigilance
QA: quality assurance
QMS: quality management system
RI: research infrastructure
SOP: standard operating procedure
UCD: University College Dublin
WG: working group

## Supporting information

Data S1. Summary of main changes related to the EU clinical trial Regulation
Data S2. Background information about ECRIN
Data S3. Questionnaire
Data S4. Some useful links

## References

[1] Atal, I. et al. (2015) “Differential globalization of industry‐ and non‐industry–sponsored clinical trials”, PLOS ONE. Edited by J. Lexchin, 10(12), p. e0145122. doi: 10.1371/journal.pone.0145122. 10.1371/journal.pone.0145122 PMC4681996 26658791

[2] OECD (2011) Facilitating International Cooperation in Non‐Commercial Clinical Trials. Available at: http://www.oecd.org/sti/inno/49344626.pdf ().

[3] EU CTR (2014) EU Clinical trial Regulation. Available at: https://ec.europa.eu/health/sites/health/files/files/eudralex/vol-1/reg_2014_536/reg_2014_536_en.pdf ().

[4] HMA CTFG (2019) EU Clinical Trials Facilitation and Coordination Group CTFG. Working group report. Available at: https://www.hma.eu/fileadmin/dateien/Human_Medicines/01-About_HMA/Working_Groups/CTFG/2019_08_EUMS_national_pilot_projects_intro___VHP-plus.pdf ().

[5] ECRIN (2019) European Clinical Research Infrastructures Network (ECRIN). Available at: https://www.ecrin.org/ ().

[6] ECRIN statutes (2013) “European Clinical Research Infrastructure Network (ECRIN). Statutes. 2013”, p. 13. Available at: https://www.ecrin.org/sites/default/files/ECRIN%20statutes/Statutes%20ECRIN%20EN.pdf.

[7] Magnin, A. et al. (2019) “European survey on national training activities in clinical research”. doi: accepted for publication. 10.1186/s13063-019-3702-z PMC6821032 31665085

[8] HRDOP (2019) Human Resource Development Operational Programme | Széchenyi 2020. Available at: https://www.palyazat.gov.hu/human_resource_development_operational_programme .

[9] SCTO platforms (2019) News ‐ scto. Available at: https://www.scto.ch/en/network/scto-platforms.html ().

[10] SUKL (2019) Czech Republic national regulatory agency, Human medicines ‐ Czech Republic/EU, State Institute for Drug Control. Available at: http://www.sukl.eu/sukl/human-medicines-czech-republic-eu ().

[11] SCTO Guidelines (2019) Guidelines ‐ scto. Available at: https://www.scto.ch/en/publications/guidelines.html ().

[12] SCTO Monitoring (2019) News ‐ scto. Available at: https://www.scto.ch/en/network/scto-platforms/monitoring.html ().

[13] Swissethics (2019) Swissethics. Available at: https://www.swissethics.ch/templates_e.html ().

[14] Norway (2019) “Call for proposals—Program for klinisk behandlingsforskning”. Available at: http://kliniskforskning.rhf-forsk.org/utlysning/utlysning/call-in-english/ ().

[15] Gazzetta ufficiale (2018) *Gazzetta Ufficiale*. Available at: https://www.gazzettaufficiale.it/eli/id/2018/1/31/18G00019/sg ().

[16] F‐CRIN (2019) Liste des formations en présentiel à la recherche clinique | *F‐CRIN*. Available at: https://www.fcrin.org/training-course-advisor/informations-referencees/les-formations ().

[17] CLIC (2013) *CLIC*. Available at: http://clic.pharmaceutical-medicine.pt/ ().

[18] Boeynaems, J.‐M. et al. (2013) “A European approach to clinical investigator training”, Frontiers in Pharmacology, 4. doi: 10.3389/fphar.2013.00112. 10.3389/fphar.2013.00112 PMC3766792 24058345

[19] Ohmann C et al. Raising standards in clinical research – The impact of the ECRIN data centre certification programme, 2011–2016. Contemporary Clinical Trials Communications. 2017;5:153‐159. 10.1016/j.conctc.2017.02.005. 29740631 PMC5936703

[20] Madeira, C. et al. (2019) “Transparency and accuracy in funding investigator‐initiated clinical trials: a systematic search in clinical trials databases”, BMJ Open, 9(5), p. e023394. doi: 10.1136/bmjopen‐2018‐023394. 10.1136/bmjopen-2018-023394 PMC6530385 31092640

[21] Gehring M et al. Factors influencing clinical trial site selection in Europe: the Survey of Attitudes towards Trial sites in Europe (the SAT‐EU Study). BMJ Open. 2013;3(11):e002957. 10.1136/bmjopen-2013-002957. PMC3831096 24240138

[22] Fukushima M et al. The global academic research organization network: Data sharing to cure diseases and enable learning health systems. Learning Health Systems. 2019;3(1):e10073. 10.1002/lrh2.10073. 31245596 PMC6508835

[23] ECRIN members (2019) European Clinical Research Infrastructure Network (ECRIN). Members & Observers | ECRIN. Available at: https://www.ecrin.org/who-we-are/members-observers ().

