# Supplementary material for: European survey on national harmonization in clinical research
Source: Learn Health Syst. 2020 Feb 21;5(2):e10220. doi: 10.1002/lrh2.10220 (PMC8051345; doi:10.1002/lrh2.10220)
Supplement: Supplementary file 1 — Data S1. Summary of main changes related to the EU clinical trial Regulation Data S2. Background information about ECRIN Data S3. Questionnaire Data S4. Some useful links [file LRH2-5-e10220-s001.docx]

**Appendix 6.3 ECRIN Scientific Partners: National activities – harmonisation (Questionnaire)**

1. **To what extent do you work on the harmonisation of clinical research processes on the *national* level?**

Please differentiate clearly between network-internal harmonization activities (of national relevance) and external harmonization activities in collaboration with other entities (authorities etc.). Activities regarding trainings are specified from point 6 on and should be listed there as well.

Please characterize each relevant activity according to the following criteria:

| Type of activity  (consultation of documents, working group, etc.) | 1 | 2 | 3 | 4 |
| --- | --- | --- | --- | --- |
| Internal / external? |  |  |  |  |
| Scope of activity (in collaboration with national bodies, within network but of national relevance, etc.) |  |  |  |  |
| External partner(s) (e.g. ethic committees, official national bodies, etc.) |  |  |  |  |
| Type of collaboration (systematic or opportunity-driven, driven by network or partner) |  |  |  |  |
| Comments / links |  |  |  |  |

Please add additional table/columns for activities 5 – x

| General remarks to question 1: |
| --- |
|  |

Please complete the table (marking with a cross as appropriate)

**Please note: table with text above and crosses in table below should be consistent, any inconsistency will trigger a query!**

| **Harmonisation activity** | **Country** | | | | | | | | | | |
| --- | --- | --- | --- | --- | --- | --- | --- | --- | --- | --- | --- |
|  | **Cze** | **Fra** | **Ger** | **Hun** | **Ire** | **Ita** | **Nor** | **Port** | **Slo** | **Spa** | **Sui** |
| Working groups |  |  |  |  |  |  |  |  |  |  |  |
| Consulting |  |  |  |  |  |  |  |  |  |  |  |
| SOPs |  |  |  |  |  |  |  |  |  |  |  |
| Templates, tools |  |  |  |  |  |  |  |  |  |  |  |
| Services |  |  |  |  |  |  |  |  |  |  |  |
| Curricula for CR staff* |  |  |  |  |  |  |  |  |  |  |  |
| Other activities |  |  |  |  |  |  |  |  |  |  |  |

* should also be reflected again under point 6ff.

1. **To what extent do you share your templates / documents/ recommendations developed in your network** (free, limited to network, etc.)**?**

Please add links to existing repositories/web pages.

1. **Do you have or offer local / national certification programs for aspects of clinical research?**(certified training programs are specified from point 6 on).

If yes, please specify according to:

- which certification
- target group (within network, external clients or organisations)

Please add links as appropriate.

1. **How would you overall rate your influence on harmonisation regarding clinical research in your country?**

The main focus is on your impact at the national level, not within your network (although this may of course have an indirect effect).

Please make the overall assessment of your impact on harmonization on the national level by ticking one of the boxes below:

| very low | low | moderate | high | very high |
| --- | --- | --- | --- | --- |
|  |  |  |  |  |

If low to very low: what are the reasons? What would be needed to get more influence?

**5. Are you perceived as facilitator for clinical researchers or rather as adding
 bureaucracy to their work?**

**ECRIN Scientific Partners: National Training activities**

**6. Please give an overview on the training activities for clinical research in your country**

Please characterize each relevant training activity according to the following criteria:

| Which training (e.g., GCP, PV, monitoring etc.) | 1 | 2 | 3 | 4 |
| --- | --- | --- | --- | --- |
|  |  |  |  |  |
| Target group (e.g. study nurses, investigators) |  |  |  |  |
| Obligation (optional, mandatory) |  |  |  |  |
| Type of training (e.g. course, summer school, master etc.) |  |  |  |  |
| Who provides the training (local, national scientific network, other – please describe) |  |  |  |  |
| Standardized curriculum (none, within national scientific network, national, other) |  |  |  |  |
| Comments / links |  |  |  |  |

Please add additional table/columns for trainings 5 – x

| General remarks to question 6: |
| --- |
| Please add relevant links as appropriate. |

Please complete the table (marking with a cross as appropriate)

**Please note: table with text above and crosses in table below should be consistent, any inconsistency will trigger a query!**

| **Training activity** | **Country** | | | | | | | | | | |
| --- | --- | --- | --- | --- | --- | --- | --- | --- | --- | --- | --- |
|  | **Cze** | **Fra** | **Ger** | **Hun** | **Ire** | **Ita** | **Nor** | **Port** | **Slo** | **Spa** | **Sui** |
| GCP |  |  |  |  |  |  |  |  |  |  |  |
| Study nurse |  |  |  |  |  |  |  |  |  |  |  |
| Investigator |  |  |  |  |  |  |  |  |  |  |  |
| Monitoring |  |  |  |  |  |  |  |  |  |  |  |
| PV/clin.pharm. |  |  |  |  |  |  |  |  |  |  |  |
| PI/study coord. |  |  |  |  |  |  |  |  |  |  |  |
| CRO operators |  |  |  |  |  |  |  |  |  |  |  |
| Methodology |  |  |  |  |  |  |  |  |  |  |  |
| QMS |  |  |  |  |  |  |  |  |  |  |  |
| Postgraduate |  |  |  |  |  |  |  |  |  |  |  |
| Other |  |  |  |  |  |  |  |  |  |  |  |

**7. Are there (national) minimal requirements for a specific function (e.g. PI, monitor, biostatistician)?**

Please add links as appropriate.

**8. Are there (national) requirements for continuous education in clinical research?**Please add links as appropriate.

**9. Is there an overarching, national strategy/roadmap/standards for training in clinical research?**

Please add links as appropriate.

**10. What are the career options in clinical research in your countries? What academic titles in clinical research are available?**

**Career options:**

**Academic titles:**

Please add links as appropriate.

**11. Would it make sense to offer (complementary) international training(s) in clinical research?**

If yes, describe which training(s) and what could be the role of ECRIN.

Please add links as appropriate.
